# A Criticism of Homœopathy

**Published:** 1909-04-10

**Authors:** 


### The Hospital

A Journal of

The Medical Sciences and Hospital Administration.

New Series. No. 110, Vol. V. [No. 1182, Vol. XLVI.] SATURDAY, APRIL 10, 1909.

### A Criticism of Homœopathy.

A meeting was lately held at the Mansion House, under the presidency of the Lord Mayor, to inaugurate a national fund in support of homœopathy; so one must suppose that this cause, like that of orthodox medicine, is pressed for funds. Homœopathy has always made a great stir for its size, and even now the word is often heard. Yet the world at large knows little of what homœopathy really stands for. Moreover, homœopaths have claimed of late that the recent advances of vaccine and serum treatments are evidence in favour of their contentions. So it may not be amiss to review the history of homœopathy and rebut the fallacy contained in this assumption.

Homœopathy is a system of therapeutics based upon the dogma "*similia similibus curentur*"—"Let similars be treated by similars." The system was given to the world in 1796 by Hahnemann, and is founded upon the belief that diseases are to be cured by drugs capable of producing in a healthy individual symptoms similar to those of the disease to be treated. Strychnia on this assumption would, we suppose, be the natural remedy for tetanus, and ether-inhalations for bronchitis. But here we speak with diffidence, not being well posted in the later glosses upon the text. In addition, Hahnemann promulgated a theory to account for all chronic diseases. They were, he said, all derived, directly or remotely, from one or other of three sources—namely, the itch, syphilis, or sycosis. This remarkable conception, it is sad to relate, had but a limited vogue, and one quite disproportionate to its monumental originality. Nevertheless, it is well to bear in mind that the originator of homœopathy was capable of producing such a conception, for homœopaths are not wont to boast about it. A third item of importance in the creed is the doctrine of minute doses. This doctrine was, we are told, a later creation of the founder, and totally distinct from the basal law quoted above, although in the minds of many it is the most prominent feature of present-day homœopathy. Lastly, Hahnemann taught that "even after the material medicinal particles of a drug have been subdivided to the fullest extent, the continuation of the dynamisation, or trituration, or succussion, develops a spiritual curative agency, and that the higher the potency the more subtle and more powerful is the curative action." Such were the tenets of the father of homœopathy. His course appears to have been dictated by a feeling of revolt against the complicated and futile concoctions which filled the pharmacopœias of his day; and we do not deny that his creed may have exercised a salutary influence upon the ignorant drug-vending which filled so large a place in the medicine of the time. Nor need we doubt his sincerity in his beliefs, for he suffered much on their account.

Few things are more remarkable than the ease with which new religions and new systems of treatment obtain a vogue, and keep it in spite of persecution, of ridicule, of demonstrable absurdity, and an infinite variety of other disadvantages. The fact is a commentary upon the credulity and conservatism of mankind in general; but it is a singular thing to see men of the world, who in the ordinary matters of life follow the dictates of reason, surrendering their judgment to a long-exploded superstition. We should not have bestowed any concern upon this business were it not for the fear that money urgently required for the prosecution of scientific medicine may be diverted, through the ignorance of benefactors, into the superstitious channels of homœopathy. It is for this reason that we wish to expose the fallacy that vaccine-therapies are of the essence of homœopathy, as supporters of the movement are fond of claiming. That the confusion should have been admitted for a moment is evidence of the uncritical faculty without which such creeds as homœopathy are doomed to early death. The fallacy in brief is this. To the homœopathist the symptom of a disease is everything, the cause nothing. What matter though convulsions may be due to tetanus or epilepsy, strychnia or uræmia? There are the convulsions, and *ex hypothesi* strychnia should cure them. On the other hand, the principle underlying vaccine therapy is that the introduction into the body of a disease-producing substance in a modified and little-virulent form enhances the protective forces of the body against more powerful and virulent examples of that particular substance. It will be seen that from this point of view the symptoms of the disease are nothing to the point, but the cause everything, since specificity is the key-note of vaccine therapies. The abiding distinction between scientific vaccine-treatment and the homœopathic principle can be made clear by an example. Both the tubercle bacillus and the bacillus coli communis may attack the bladder and give rise to symptoms of cystitis. Scientific medicine says that the appropriate vaccine for tuberculous cystitis is one prepared from tubercle bacilli, and for the other variety one prepared from the corresponding organism. But the creed of homœopathy would justify its practitioners in classing both varieties together and treating them indifferently with tuberculin or bacillus coli vaccine, regardless of the nature of the disease, and merely on the score that the symptoms are alike.

The ease with which even astute minds surrender their judgment and allow themselves the luxury of loose thinking must be generally recognised from the attendance at the Mansion House meeting of men with a public history.

